# A PMM2-CDG caused by an A108V mutation associated with a heterozygous 70 kilobases deletion case report

**DOI:** 10.1186/s13052-022-01355-x

**Published:** 2022-10-11

**Authors:** E. Lebredonchel, A. Riquet, D. Neut, F. Broly, G. Matthijs, A. Klein, F. Foulquier

**Affiliations:** 1 UMR 8576, Univ. Lille, CNRS, UGSF - Unité de Glycobiologie Structurale Et Fonctionnelle, 59000 Lille, France; 2 Centre de Biologie Et Pathologie, Lille Medical Center, University of Lille, UAM de glycopathologies, 59000 Lille, France; 3 Lille University Hospital Center Paediatrics, 59000 Lille, France; 4 Cabinet de Pédiatrie, 93 Rue de la Paix, 62200 Boulogne-sur-Mer City, France; 5 Centre Hospitalier Régional Universitaire de Lille Centre de Biologie Pathologie, 59000 Lille, France; 6 Laboratory for Molecular Diagnosis, Center for Human Genetics, 3000 Leuven, KU Belgium

**Keywords:** CDG, PMM2, Deletion mutation, PMM2-CDG, Whole genome sequencing

## Abstract

**Background:**

Congenital Disorders of Glycosylation (CDG) are a large group of inborn errors of metabolism with more than 140 different CDG types reported to date (1). The first characterized, PMM2-CDG, with an autosomal recessive transmission, is also the most frequent. The *PMM2* gene encodes a phosphomannomutase. Here, a novel genetic variation causing PMM2-CDG is reported.

**Case presentation:**

We report the case of a French child, from healthy and unrelated parents, presenting congenital ataxia with hypotonia, hyperlaxity, inverted nipples, as well as altered coagulation parameters and liver function. Transferrin isoelectrofocusing revealed a typical type I CDG profile. Direct Sanger sequencing and quantitative PCR of *PMM2* revealed a unique and novel genotype. On one allele, the patient was heterozygote with a known missense variant NM_000303.3(PMM2):c.323C > T, p.Ala108Val in exon 4. On the second allele, whole genome sequencing (WGS) indicated the presence of a novel heterozygous 70 kb deletion.

**Conclusion:**

We report in the present paper the largest known heterozygous deletion of a *PMM2* gene. The observation reveals the impact of a precise diagnostic on genetic counselling: by using WGS, an erroneous conclusion of homozygosity in the case of a relatively rare variant could be avoided, and an index patient with healthy and unrelated parents correctly identified.

## Background

Congenital Disorders of Glycosylation (CDG) are a rapidly expanding family of genetic diseases. Today, 140 different CDG subtypes have been reported [1] categorized in 2 groups: CDG-I, affecting steps before the oligosaccharide precursor transfer in the endoplasmic reticulum, and CDG-II, affecting the steps following the transfer, mostly in the Golgi apparatus. The first patient cases were reported 40 years ago by Jaeken et al., [2]. Mutations in *PMM2* (OMIM 601,785), a gene on chromosome 16p13 encoding a phosphomannomutase were shown to be responsible for the disease. This enzyme catalyzes the conversion in the cytosol of mannose-6-P to mannose-1-P, necessary for the synthesis of donor substrates for glycosylation, GDP-mannose, and Dol-P-mannose.

First named CDG-Ia then changed into PMM2-CDG in 2009 [3], the disease is thought to represent 70% of the total CDG cases, with an estimated incidence around 1:20 000 [4]. The spectrum of clinical phenotypes and severity is broad and is characterized with mainly a psychomotor development impairment associated with cerebellar hypoplasia, hypotonia, dysmorphia, and coagulopathy [5]. The lethality rate in the first 4 years of life is about 20%. Beyond childhood, PMM2-CDG patients have a good life expectancy [6]. The number of *PMM2* mutations classified in HGMD (https://my.qiagendigitalinsights.com) is 142, most of these, 113, are missense mutations and the most frequent is p.Arg141His (R141H) [7]. We report the particular genotype of a PMM2-CDG patient with a heterozygous p.Ala108Val (A108V) mutation on one allele and a first-described > 70 kb-deletion on the other allele.

## Case presentation

We report the case of a PMM2-CDG French child of Caucasian descent. She was born at term with a length of 48 cm for 3.110 kg, after uncomplicated pregnancy with vaginal delivery. The AGPAR score was 10 both at 1 min and 5 min and the newborn screening results were normal. The parents are unrelated and the 6-years older brother is healthy. Parents reported abnormal abrupt movements of the child after birth that stopped spontaneously.

Biologically, at 9 months of age, elevated transaminases TGO = 50 UI/ L, TGP = 51 [normal values 10–35 UI/ L] and normal coagulation parameters with ATIIIA = 63% [normal values 80–120%] and FXI 53% at the lower limit [normal values 50–150%] were assayed. The cytology and the thyroid function were normal.

**Fig. 1:**
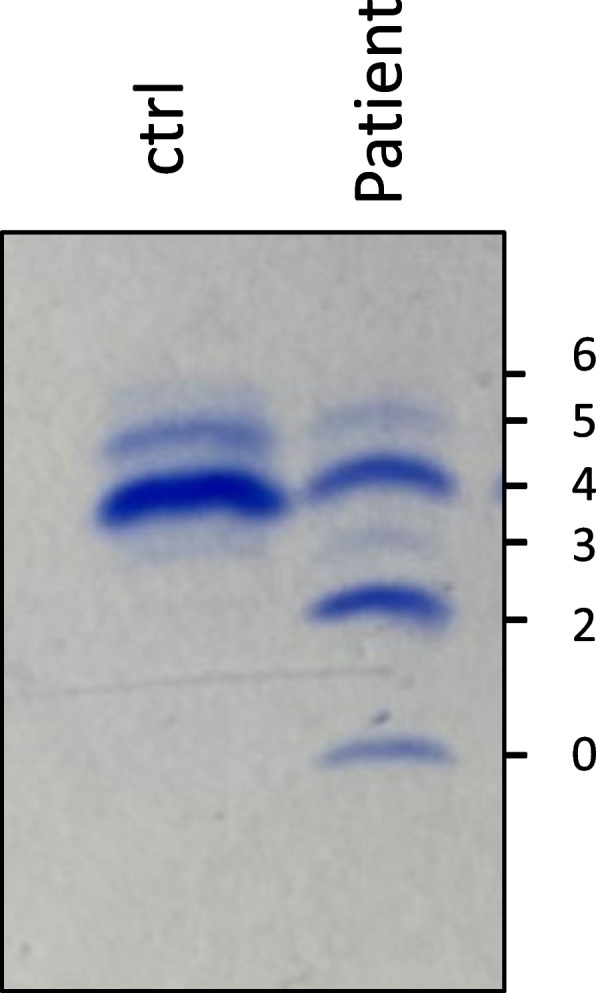
Distribution of transferrin glycoforms via transferrin isoelectrofocusing of the patient compared to a control. Numbers 0, 2, 3, 4, 5, and 6 indicate the migration position of the asialo-, disialo-, trisialo-, tetrasialo-, pentasialo-, and hexasialotransferrin forms respectively

The clinical examination at 9 months of age revealed ataxia, hypotonia, hyperlaxity, strabismus, esotropia, feeding difficulties, and inverted nipples. The child was calm and exclusively breastfed with an absence of facial dysmorphia and no sleeping disorders. At that time, the girl presented an inability to reach a seated posture. The diagnosis of CDG was oriented by an abnormal pattern in serum transferrin isoelectrofocusing [8] with an elevation of asialo- and disialo-transferrin, typical from a type I CDG (Fig. 1). Brain MRI revealed cerebellar abnormalities with vermis hypoplasia. The child finally reaches a seated posture at 11 months of age.

## Genetic testing

**Fig. 2:**
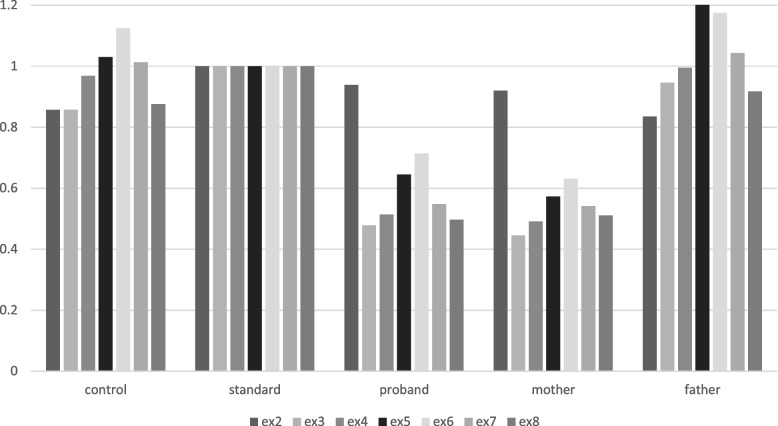
PCR quantification of *PMM2* exons. qPCR of exons 2 to 8 of PMM2 in the proband and the parents compared to standard and control samples

**Fig. 3:**
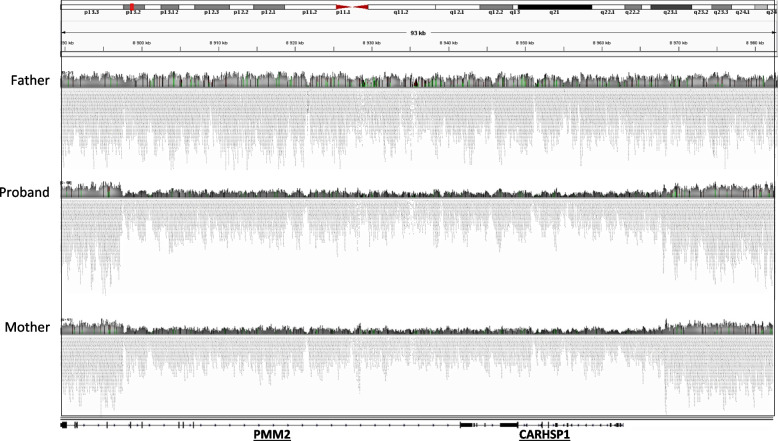
Deletion breakpoint localization. Reads quantification in position chr16:8,897,826–8,968,278 of the family members. A deletion of 70453pb is seen for the proband and the mother. The genes affected by the deletion are named on the bottom of the figure

Direct Sanger sequencing of the 8 exons of *PMM2* reported a seemingly homozygous variant rs200203569 NM_000303.3(PMM2): c.323C > T in exon 4. The variant leads to a missense substitution of alanine 108 to proline (p.Ala108Pro), commonly named A108V, known to be pathogenic (ClinVar, SIFT, Mutation Taster). The A108V mutation is quite rare and is often associated with R141H, the most common deleterious *PMM2* mutation, in compound heterozygous patients [9]. A homozygous presentation of R141H variant is thought to be incompatible with life as no case was reported so far [10]. For the A108V variant, the gnomAD (2.1) website reports a frequency of 0.0012% in the overall population. To our knowledge, no homozygous A108V patient is reported in the literature and, as the parents were unrelated, further genetic explorations were conducted. Direct Sanger sequencing of *PMM2* of the paternal DNA reported a heterozygous A108V mutation while no mutation was found in the mother. At the time of the genetic exploration, the seemingly second variant could have been either due to the de novo variant or to the absence of sequence at the same location on the other allele. A quantitative PCR (qPCR) of the 13 exons was performed, showing a reduction of the DNA of the gene by 50% in the mother and the proband from exon 3 to exon 8, the last exon of *PMM2* (Fig. 2). The hypothesis of the de novo variant was rejected as the heterozygous deletion in *PMM2* gene was found. To evaluate the extent of the deletion that goes beyond *PMM2* gene, Whole Genome Sequencing (WGS) was performed. WGS was preferred to CGH array to accurately determine the exact position of the breakpoints. WGS allowed to delineate the deletion of 70,453 bp in position chr16:8,897,826–8,968,278 (Fig. 3). In the HGMD database, the largest deletion reported is 28 kb-long.

The deletion also affects a part of *CARHSP1* (Calcium Regulated Hear Stable Protein 1) (OMIM: 616,885) gene that plays a role in TNF mRNA stabilization, seemingly not affecting the phenotype.

## Discussion and conclusions

In the present study, we described the case of a PMM2-CDG patient with congenital ataxia. The genotype identified in the child is novel. The clinical course was relatively mild for a child with PMM2-CDG as the child does not present facial dysmorphia. Given that the maternal mutation could not be detected upon Sanger sequencing, further investigations were performed to precise the genetic transmission of the disease.

A108V mutation was first described in France [7] and the effect on the enzyme activity is unknown. When associated to a mutation in R141H the remaining phosphomannomutase activity in leucocytes is 0.09% [11].

Quantitative PCR and WGS allowed to identify a large deletion on the maternal allele. A new deletion of 70,453 bp in position chr16:8,897,826–8,968,278 could be accurately detected with WGS including 6 exons of *PMM2* and a part of *CARHSP1* gene. Knockout of CARHSP1 has demonstrated the role of CARHSP1 as a TNF-α mRNA stability enhancer [12]. GnomAD database reports various loss of function heterozygote mutation for CARHSP1, indicating that the observed pathology is mainly due to the phosphomannomutase defect.

To our knowledge, this is the largest *PMM2* deletion reported so far. Our example illustrates the usefulness of WGS in the case of an apparent homozygous variant in an unrelated family. Wherever possible, compound heterozygosity has to be confirmed with a parental genetic study. As the disease transmission for the couple is 25%, an antenatal diagnostic can now be proposed for future pregnancies.

This case underlines the importance of the correlation between the phenotype description and the genetic study, especially in disorders with a wide phenotypic spectrum like PMM2-CDG [13], where it is essential for a correct diagnosis to search thoroughly over the appearances.

In case of strong suspicion of PMM2-CDG based on the clinical phenotypes and despite the absence of transferrin abnormalities, the molecular analysis should be performed to avoid any diagnostic deadlocks.

## Data Availability

The datasets used and/or analysed during the current study are available from the corresponding author on reasonable request.

## Abbreviations

CDG: Congenital Disorder of Glycosylation
PMM2: Phosphomannomutase 2
WGS: Whole Genome Sequencing
Bp: Base pair
